# Impact of Navigation Assistance on Perioperative Outcomes in Sacroiliac Joint Arthrodesis: A Comparative Study

**DOI:** 10.7759/cureus.95751

**Published:** 2025-10-30

**Authors:** Attila Sarkadi, Adolf Mueller, Vitaly Sokotukhin, Stephan Lackermair, Eric O Sarpong, Hannes Egermann

**Affiliations:** 1 Neurosurgery, Klinikum Barmherzige Brüder Regensburg, Regensburg , DEU; 2 Neurosurgery, Klinikum Barmherzige Brüder Regensburg, Regensburg, DEU

**Keywords:** diana implant, fluoroscopy time, image-guided surgery, minimally invasive spine surgery, navigation, operative efficiency, radiation exposure, sacroiliac joint arthrodesis

## Abstract

Background

Navigation systems are increasingly applied to enhance precision and reduce radiation exposure in sacroiliac joint (SIJ) arthrodesis. However, evidence comparing navigated and conventional procedures remains limited. This study evaluated perioperative outcomes of navigation-assisted versus non-navigated SIJ arthrodesis using the DIANA implant system (Signus GmbH, Alzenau, Germany).

Methods

A retrospective comparative study was conducted on 30 patients who underwent SIJ arthrodesis between 2011 and 2021 at the Department of Neurosurgery, Hospital of the Order of Merciful Brothers, Regensburg, Germany. Thirteen patients received navigation-assisted surgery and 17 underwent conventional fluoroscopy-guided procedures. Perioperative parameters assessed included operative time, radiation dose, fluoroscopy time, and intraoperative blood loss. Group comparisons were performed using independent-samples t-tests and one-way ANOVA, with significance set at p < 0.05.

Results

Baseline characteristics (age, sex, BMI) were comparable between groups. Mean operative time was slightly shorter with navigation (146 ± 37 min) versus non-navigated procedures (162 ± 31 min; p = 0.198). Radiation dose was lower in the navigation group (718 ± 379 mGy·cm² vs 1015 ± 532 mGy·cm²; p = 0.100), though not statistically significant. Fluoroscopy time was significantly reduced with navigation (82 ± 40 s vs 112 ± 34 s; p = 0.037). Blood loss was similar between groups (246 ± 125 mL vs 238 ± 110 mL; p = 0.855).

Conclusion

Navigation in SIJ arthrodesis significantly decreases fluoroscopy time while maintaining similar operative duration, radiation dose, and blood loss compared to conventional techniques. Its main advantage lies in improving imaging efficiency and workflow precision. Larger prospective studies are warranted to validate these findings and assess long-term clinical outcomes.

## Introduction

Low back pain (LBP) is the leading cause of years lived with disability worldwide, with a lifetime prevalence exceeding 70% in industrialized countries and an estimated point prevalence of 9-12% globally [1]. Beyond individual suffering, LBP has a profound economic impact, with annual healthcare expenditures and productivity losses totaling billions of dollars. The sacroiliac joint (SIJ) is increasingly recognized as a significant contributor to this burden, accounting for 15-30% of chronic, non-radicular LBP cases in specialized pain clinics [2]. SIJ dysfunction is also a notable cause of persistent pain following lumbar fusion, as altered spinal biomechanics and increased stress transmission across the pelvis can accelerate SIJ degeneration [3,4]. Such secondary SIJ pathology is reported in up to 40% of patients with continued low back pain after fusion surgery, underscoring the need for careful evaluation in this population [3].

Initial management is conservative, including targeted physical therapy, intra-articular steroid injection, and radiofrequency ablation. While some patients experience short-term relief, long-term durability remains limited, with recurrence rates commonly exceeding 50% within one year [5,6]. For carefully selected patients with confirmed SIJ-mediated pain refractory to non-operative measures, surgical fusion has long been established as a definitive treatment option [7]. The last decade has witnessed the rapid adoption of minimally invasive SIJ fusion (MIS-SIJF) systems, which have expanded utilization by offering smaller incisions, shorter recovery, and reduced perioperative morbidity compared to open techniques. Registry and post-market surveillance data support their safety, showing low complaint rates and favorable implant survivorship up to five years, though device malposition and patient selection remain critical considerations [8,9].

Parallel advances in intraoperative imaging and navigation technologies aim to enhance precision during pelvic fixation and SIJ fusion. Intraoperative CT with navigation has been associated with improved implant placement accuracy and reduced fluoroscopy requirements in sacropelvic procedures [10]. These developments warrant comparative evaluation of navigated versus non-navigated SIJ arthrodesis, with emphasis on operative efficiency and radiation exposure. This study aimed to compare perioperative outcomes between navigation-assisted and conventional SIJ arthrodesis, focusing on operative time, radiation exposure, fluoroscopy duration, and intraoperative blood loss. The objective was to assess whether navigation improves workflow efficiency and imaging parameters without compromising surgical safety.

## Materials and methods

Study design and setting

We conducted a retrospective comparative study of patients who underwent sacroiliac joint (SIJ) arthrodesis at the Department of Neurosurgery, Hospital of the Order of Merciful Brothers (Krankenhaus Barmherzige Brüder), Regensburg, Germany, between January 2011 and December 2021. The primary objective was to evaluate differences in operative efficiency and perioperative outcomes between procedures performed with and without intraoperative navigation.

Study population

Thirty consecutive patients who underwent SIJ arthrodesis with the DIANA implant system (Signus GmbH, Alzenau, Germany) were included. Patients were divided into two groups based on the surgical technique employed: procedures performed with navigation (n = 13) and procedures performed without navigation (n = 17). Baseline variables collected included age, sex, and body mass index (BMI). Patients with incomplete clinical records were excluded.

Surgical technique

Navigated and Non-Navigated Procedures

Sacroiliac joint (SIJ) arthrodesis in this series was performed either with or without intraoperative navigation. Navigation-assisted procedures employed modern neuronavigation equipment to provide real-time three-dimensional guidance for trajectory control, while non-navigated procedures relied solely on conventional fluoroscopy. This distinction enabled direct comparison of the two approaches and facilitated evaluation of potential advantages of navigation in terms of accuracy, operative efficiency, and radiation exposure.

Clinical Procedure

**Figure 1:**
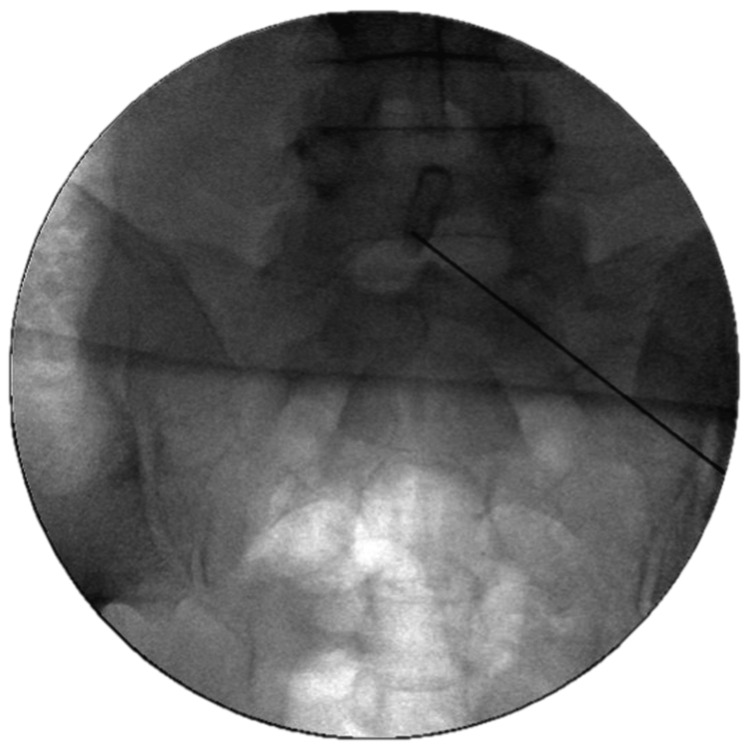
Skin incision marking under fluoroscopic guidance to delineate the upper sacral margin This intraoperative image demonstrates the marking of the upper sacral margin using a metallic guidewire under fluoroscopy before skin incision. This step ensures accurate localization of the sacroiliac joint (SIJ) and correct orientation for DIANA implant (Signus GmbH, Alzenau, Germany) placement.

All patients underwent arthrodesis using the DIANA implant system (Signus GmbH, Alzenau, Germany). Following endotracheal intubation, patients were placed in the prone position on a radiolucent carbon operating table. The image converter was adjusted in anteroposterior, lateral, and oblique planes. Under fluoroscopic guidance, the skin incision was marked with a wire to delineate the upper sacral margin (Figure 1). After standard preparation and draping, a midline skin incision approximately 7 cm in length was made. Subcutaneous tissues were dissected along the fascia up to the posterior superior iliac spine and the recess of the SIJ.

Neuronavigation-Assisted Exposure

**Figure 2:**
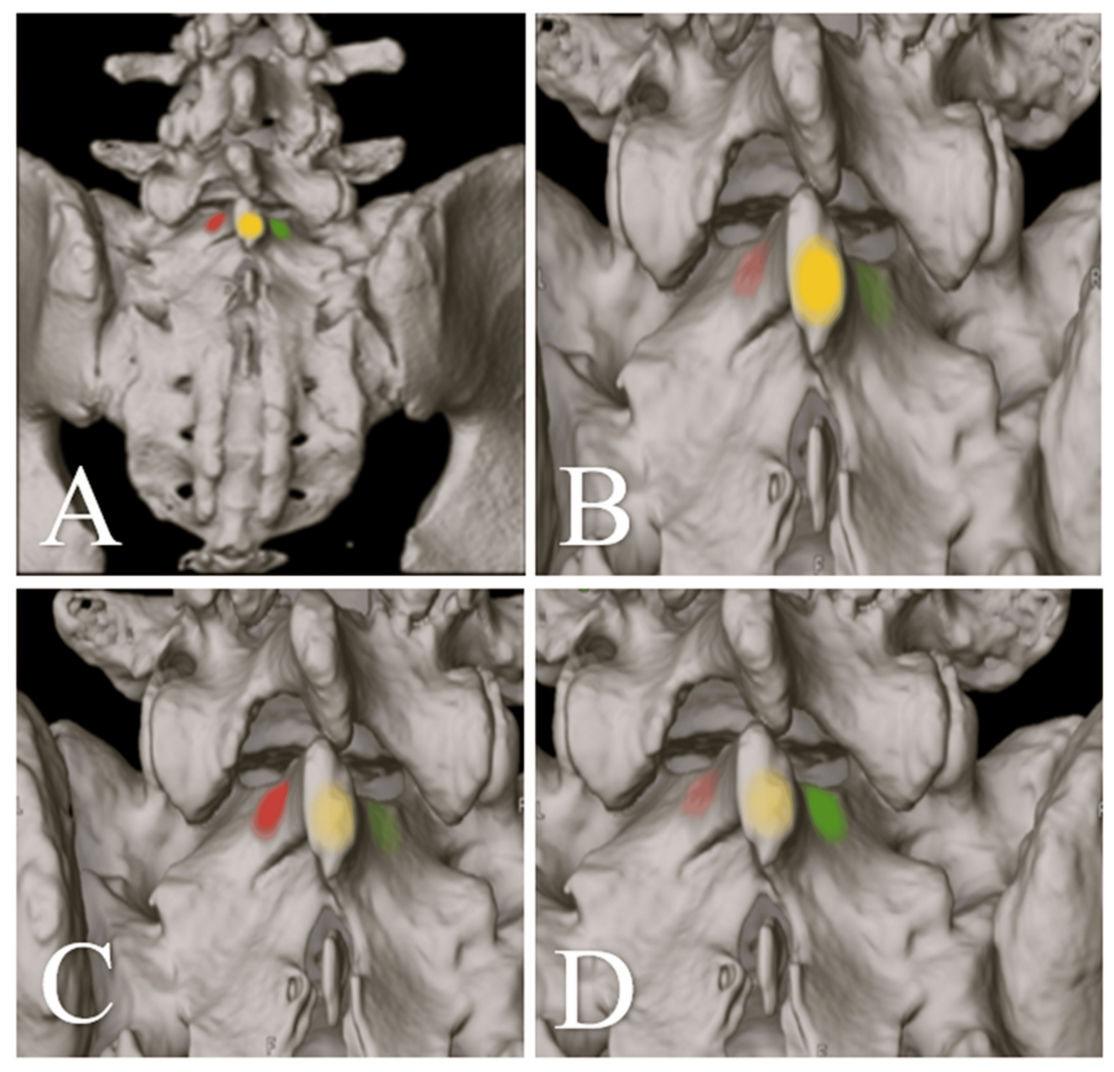
Referencing of the spinal navigation system on the Lamina of S1 Three-dimensional intraoperative reconstructions illustrating step-by-step registration of the spinal navigation system during sacroiliac joint (SIJ) arthrodesis. A. Posterior overview showing bilateral sacral anatomy with colored reference zones for orientation, B. Close-up view highlighting placement of the navigation reference marker (yellow) on the S1 lamina and identification of the sacral margins (red, green), C. Verification of alignment between virtual and anatomic landmarks during real-time neuronavigation calibration, D. Final confirmed registration demonstrating stable tracking and accurate correspondence of surface anatomy with the navigation model.

For navigated procedures, the fascia was incised along the midline over S1-S2, and paraspinal muscles were mobilized laterally to expose the lamina of S1. Accurate referencing and registration of the navigation system were performed at this site (Figure 2). The fascia over the SI joint was then incised 2-3 cm medial to the posterior superior iliac spine, and the joint recess was exposed. The dorsal sacroiliac ligaments were removed with grasping forceps and punches until the recess was visualized. The joint was prepared by reaming the bone surface, opening the SI joint with a cutting stream, and decorticating the adjacent ilium and sacral bone using a rose burr. The prepared site was packed with cancellous allograft chips and compacted with a pusher.

**Figure 3:**
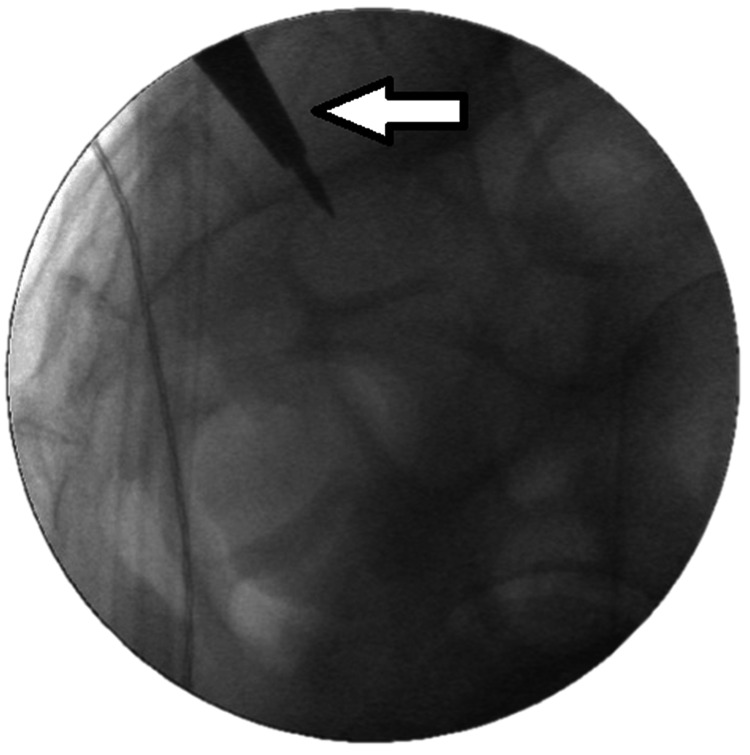
K-Wire placement with white arrow indication and distraction using sequential helix screws under navigation guidance Fluoroscopic intraoperative image showing the K-wire (white arrow) inserted through the ilium under real-time navigation control to establish the implant trajectory for sacroiliac joint (SIJ) arthrodesis. The image illustrates accurate wire positioning before sequential distraction and DIANA implant (Signus GmbH, Alzenau, Germany) insertion, confirming the precision achieved with navigated guidance.

**Figure 4:**
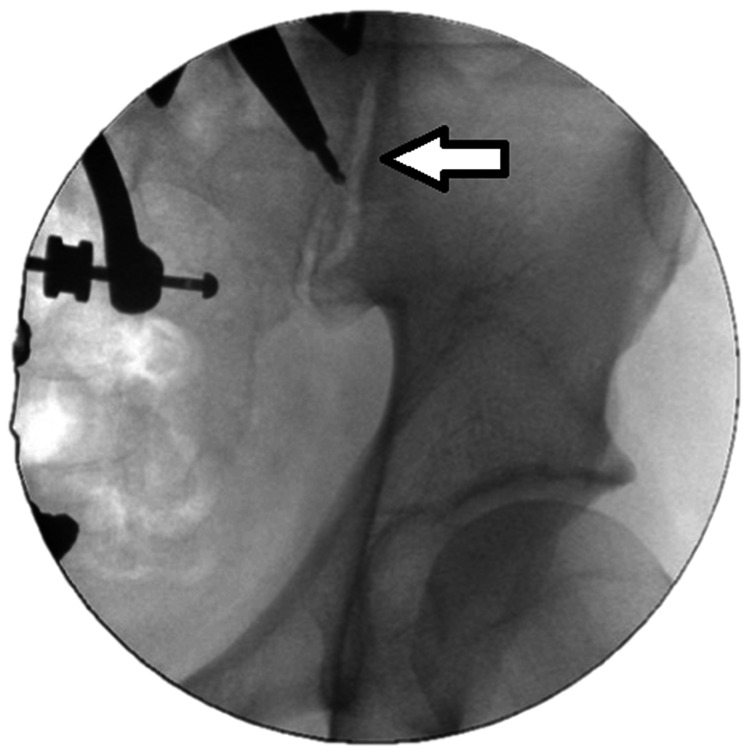
Insertion of the DIANA implant with white arrow indicating the implant trajectory Intraoperative fluoroscopic view showing the DIANA implant (Signus GmbH, Alzenau, Germany) being advanced into the prepared sacroiliac joint recess. The white arrow highlights the trajectory of the implant during placement, confirming correct orientation before final seating under navigation and fluoroscopic control.

*Implant Insertion With Navigation Guidance* Navigation allowed precise control of implant trajectory in three dimensions. The navigated drill sleeve (inner diameter 3.2 mm) guided K-wire placement into the ilium, which was confirmed by fluoroscopy in multiple planes (anteroposterior, lateral, oblique). Distraction was achieved by sequentially inserting helix screws of increasing diameter (13-21 mm) until sufficient tension was obtained. The corresponding wedge implant was then driven over the guidewire into the prepared recess, securing distraction. Reaming was performed to the anterior sacral margin, after which the definitive DIANA implant was inserted and positioned with the placement instrument (Figures 3, 4). Final fluoroscopic imaging confirmed optimal implant placement.

Implant Placement and Closure

**Figure 5:**
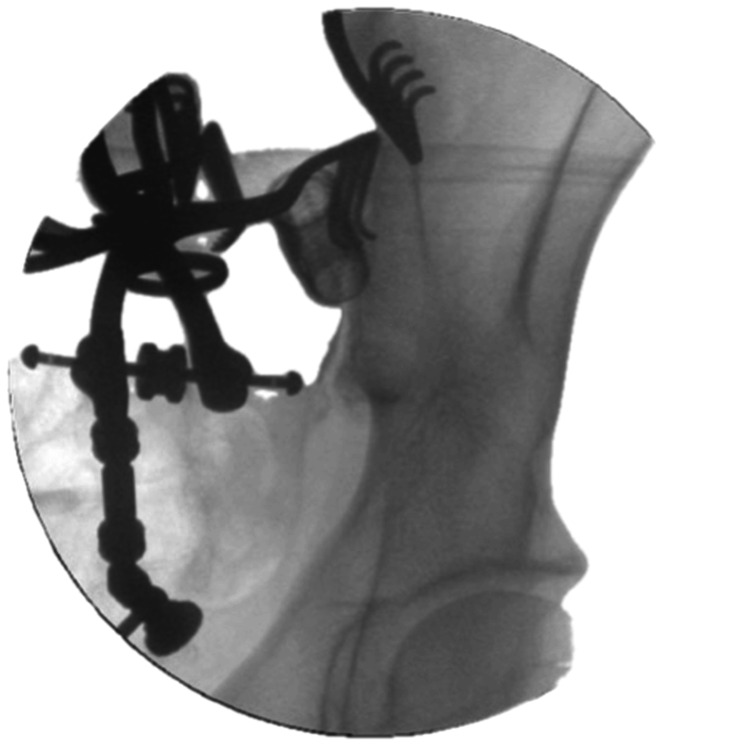
Insertion of the DIANA implant into the prepared recess Intraoperative view showing the DIANA implant (Signus GmbH, Alzenau, Germany) being advanced into the sacroiliac joint recess with the placement instrument and guide pin, and positioned to its final location under navigation and fluoroscopic control.

After implant insertion, the navigation pointer was used to reconfirm the intended trajectory (Figure 5). The residual cavity around the implant was filled with cancellous bone and compacted. Subfascial drainage was placed, and layered closure of fascia, subcutaneous tissue, and skin was performed, followed by application of a sterile dressing.

Postoperative management

**Figure 6:**
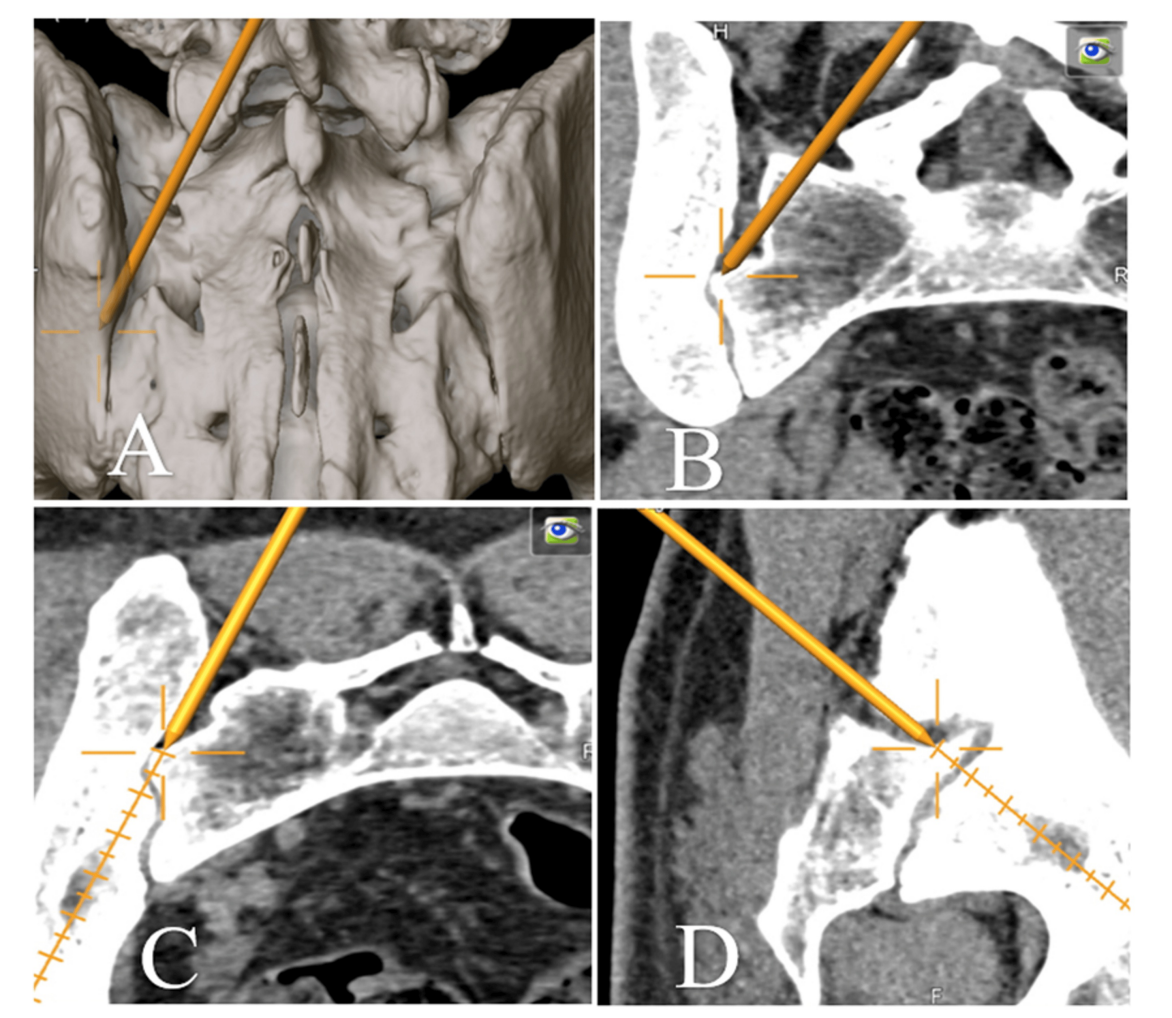
Postoperative imaging confirming final position of the DIANA implant Sequential intraoperative navigation and multiplanar CT images showing accurate implant trajectory during sacroiliac joint (SIJ) arthrodesis using the DIANA system (Signus GmbH, Alzenau, Germany). A. Three-dimensional navigation view illustrating the planned implant path from the ilium toward the sacral recess, B. Axial CT slice demonstrating correct entry point and depth of the navigated drill trajectory through the ilium, C. Coronal CT reconstruction confirming alignment of the trajectory across the SI joint at the intended depth, D. Sagittal CT view verifying final orientation of the DIANA implant path within the sacral bone window.

Postoperative CT or conventional radiography was performed before mobilization to verify implant position (Figure 6). Partial weight-bearing of up to 20 kg with crutches was advised for 8-12 weeks. Thromboprophylaxis with subcutaneous certoparin (0.3 mL daily) was administered during this period. Patients also received calcium and vitamin D supplementation to promote bone healing. Activities involving hip abduction, bending, or heavy lifting were restricted for six months to reduce the risk of implant migration or complications.

Outcome measures

The perioperative outcomes assessed included operative time, defined as the interval from skin incision to closure; radiation dose, expressed as dose-area product (DAP, mGy·cm²) as recorded by intraoperative fluoroscopy; fluoroscopy time, defined as the cumulative exposure duration in seconds; and intraoperative blood loss, measured in milliliters from suction canisters and surgical sponges, corrected for irrigation volume.

Statistical analysis

Continuous variables were summarized as mean ± standard deviation (SD) or median with interquartile range (IQR), depending on data distribution. Baseline differences (age and BMI) between groups were analyzed using independent-samples t-tests, or the Mann-Whitney U test when normality assumptions were not met. Categorical variables (sex) were compared using the Chi-square test.

Group comparisons for operative time, radiation dose, fluoroscopy time, and blood loss were performed using independent-samples t-tests. Effect sizes (Cohen’s d with 95% confidence intervals) were calculated to assess clinical relevance. A p-value < 0.05 was considered statistically significant. All analyses were conducted using Microsoft Excel 2019 (Microsoft Corp., Redmond, WA, USA) for descriptive statistics and hypothesis testing.

Ethical considerations

Written informed consent was obtained from all patients before surgery for the use of anonymized data and clinical images for research and publication. All procedures were performed in accordance with the ethical standards of the Declaration of Helsinki. Patient confidentiality was maintained at all times.

## Results

Patient distribution and demographics

**Figure 7:**
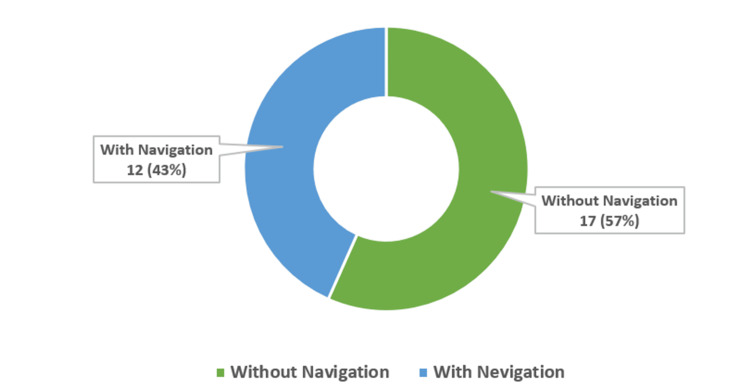
Donut chart distribution of sacroiliac joint arthrodesis with and without navigation Distribution of the study cohort (N = 30) showing 13 cases (43%) performed with navigation and 17 cases (57%) without navigation.

A total of 30 patients underwent sacroiliac joint (SIJ) arthrodesis between 2011 and 2021 and were included in the final analysis. Of these, 13 patients (43%) underwent navigation-assisted procedures, and 17 patients (57%) underwent conventional fluoroscopy-guided arthrodesis (Figure 7). The overall cohort included 18 males (60%) and 12 females (40%), with a mean age of 51.6 ± 8.9 years.

Baseline characteristics

The two groups were demographically comparable (Tables 1, 2). The mean age in the navigation group was 51.6 ± 8.9 years compared with 51.7 ± 7.5 years in the non-navigation group (p = 0.983). Gender distribution was similar, with males representing eight of 13 patients (61.5%) in the navigation group and 10 of 17 patients (58.8%) in the non-navigation group (p = 0.880). Body mass index (BMI) also did not differ significantly between groups (29.0 ± 5.4 kg/m² vs 31.2 ± 13.7 kg/m²; p = 0.585). These findings confirm that any observed differences in operative outcomes were unlikely to be influenced by baseline demographic variability.

**Table 1:** Comparison of age distribution by gender and navigation type in sacroiliac joint arthrodesis procedures (N = 30) This table summarizes the age distribution across gender and navigation groups among all 30 patients who underwent sacroiliac joint (SIJ) arthrodesis. Mean, minimum, and maximum ages are shown for males and females within both the navigation (n = 13) and non-navigation (n = 17) groups. These data confirm age comparability between groups prior to outcome analysis, indicating the absence of age-related bias.

Type of Navigation	Gender	Age			
		Count	Mean	Minimum	Maximum
Without Navigation	Male	10	50	41	56
	Female	7	54	43	73
With Navigation	Male	8	50	40	65
	Female	5	54	41	60
		Total (N)= 30	Avg= 51.60		

**Table 2:** Baseline characteristics of patients undergoing sacroiliac joint arthrodesis with and without navigation This table presents demographic variables (age, sex, and body mass index [BMI]) for both groups: navigation (n = 13) and non-navigation (n = 17). Continuous variables are expressed as mean ± standard deviation (SD), and categorical data as number (percentage). P-values were derived using independent-samples t-tests for continuous data and Chi-square tests for categorical comparisons. No significant baseline differences were found, confirming demographic homogeneity between groups.

Variable	With Navigation	Without Navigation	P-value
Age, years (mean ± SD)	51.6 ± 8.9 (N=13)	51.7 ± 7.5 (N=17)	0,983
Gender, n (%)			0,8804
- Male	8 (61.5%, N=13)	10 (58.8%, N=17)	
- Female	5 (38.5%, N=13)	7 (41.2%, N=17)	
BMI, kg/m² (mean ± SD)	29.0 ± 5.4 (N=13)	31.2 ± 13.7 (N=17)	0,585

Operative time and intraoperative parameters

The mean operative time was 146 ± 37 minutes in the navigation group and 162 ± 31 minutes in the non-navigation group. Although procedures with navigation tended to be shorter, the difference did not reach statistical significance (Tables 3, 4). Clinically, the variation of approximately 16 minutes between groups suggests only a modest potential time-saving effect.

**Table 3:** Comparison of perioperative outcomes between navigation-assisted and conventional sacroiliac joint arthrodesis (N = 30) This table presents mean, minimum, maximum, and standard deviation values for operative time, radiation dose (Dose Area Product (DAP), mGy·cm²), fluoroscopy time (seconds), and intraoperative blood loss (mL) across both groups. Independent-samples t-tests were used to compare group means, with p < 0.05 considered statistically significant.

Variables	Navigation							
	Without Navigation				With Navigation			
	Mean	Minimum	Maximum	Standard Deviation	Mean	Minimum	Maximum	Standard Deviation
OP Time	162	102	220	31	146	95	222	37
X Ray Dose (mGy·cm²)	1014.8	344.2	2253.0	531.7	718.4	384.3	1738.0	379.3
X Ray - Time (seconds)	112.18	57.00	162.00	34.40	82.23	39.00	168.00	40.21
Blood loss	238.24	100.00	500.00	109.73	246.15	100.00	500.00	124.94

**Table 4:** One-way ANOVA results for perioperative outcomes This table summarizes the analysis of variance (ANOVA) comparing operative time, radiation dose (Dose Area Product (DAP), mGy·cm²), fluoroscopy time (seconds), and intraoperative blood loss (mL) between navigation and non-navigation groups. The table reports the sum of squares, degrees of freedom (df), mean square, F-statistic, and p-values. A p < 0.05 was considered statistically significant. Among the assessed parameters, fluoroscopy time showed a significant between-group difference (p = 0.037), indicating greater imaging efficiency in the navigation group.

Variable	Source	Sum of Squares	df	Mean Square	F (p-value)
Operative Time	Between Groups	1981.995	1	1981.995	1.737 (p=0.198)
	Within Groups	31940.172	28	1140.720	
	Total	33922.167	29		
Radiation Dose (mGy·cm²)	Between Groups	647081.712	1	647081.712	2.899 (p=0.100)
	Within Groups	6249496.761	28	223196.313	
	Total	6896578.473	29		
Fluoroscopy Time (seconds)	Between Groups	6606.022	1	6606.022	4.824 (p=0.037)*
	Within Groups	38340.778	28	1369.314	
	Total	44946.800	29		
Blood Loss (mL)	Between Groups	461.916	1	461.916	0.034 (p=0.855)
	Within Groups	379954.751	28	13569.813	
	Total	380416.667	29		

Fluoroscopy duration and radiation dose

Radiation dose exposure was lower in the navigation group (718 ± 379 mGy·cm²) compared with the non-navigation group (1015 ± 532 mGy·cm²), although the reduction did not reach statistical significance (Tables 3, 4). Despite the lack of significance, this downward trend may have clinical relevance, as reducing cumulative radiation exposure remains valuable in surgical practice.

**Figure 8:**
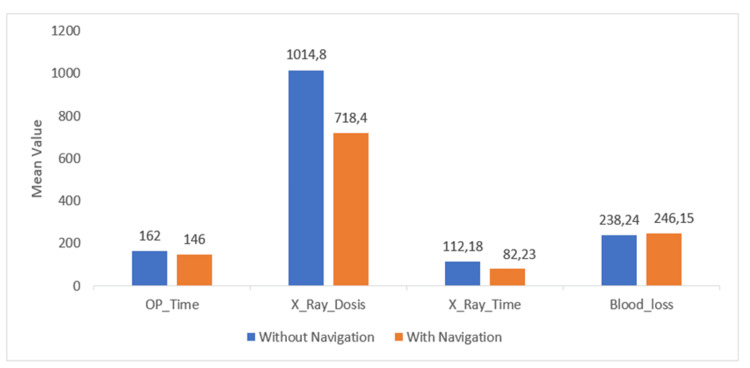
Comparative analysis of perioperative parameters between navigation and non-navigation groups This bar chart compares the mean operative time (minutes), radiation dose (Dose Area Product (DAP), mGy·cm²), fluoroscopy time (seconds), and intraoperative blood loss (mL) between groups. Y-axis: mean value for each variable. The navigation group showed a significant reduction in fluoroscopy time (p = 0.037), while other parameters remained comparable.

A significant difference was observed in fluoroscopy time. The navigation group required 82 ± 40 seconds compared with 112 ± 34 seconds in the non-navigation group, representing a 27% reduction (Figure 8). This finding indicates that navigation contributed to improved efficiency in image acquisition and reduced intraoperative radiation exposure time, supporting the hypothesis that navigational guidance enhances workflow precision.

Intraoperative blood loss

Blood loss was similar between groups, with 246 ± 125 mL in the navigation group versus 238 ± 110 mL in the non-navigation group (Tables 3, 4). These findings suggest that navigation did not influence intraoperative bleeding risk. One-way ANOVA was performed to validate outcome comparisons. No significant differences were found for operative time, radiation dose, or blood loss. The only statistically significant parameter was fluoroscopy time, which was shorter in the navigation group (Table 4). Overall, navigation-assisted SIJ arthrodesis demonstrated a significant reduction in fluoroscopy time compared with conventional techniques. Operative duration, radiation dose, and intraoperative blood loss showed no significant differences between groups, though trends toward shorter operative times and lower radiation doses with navigation were observed. These findings suggest that navigation primarily benefits intraoperative imaging efficiency without markedly altering other perioperative parameters.

## Discussion

This retrospective analysis assessed the influence of navigation on perioperative parameters during sacroiliac joint (SIJ) arthrodesis. Navigation significantly reduced fluoroscopy time, while operative time, radiation dose, and intraoperative blood loss were not statistically different. These results emphasize that navigation primarily enhances imaging efficiency rather than drastically shortening total procedure duration or reducing blood loss. The most notable finding was the significant reduction in fluoroscopy exposure. Navigation provides precise, real-time three-dimensional orientation, reducing the need for repeated radiographic verification. Similar findings have been reported in image-guided spinal instrumentation, where navigation decreases fluoroscopic checks and cumulative exposure for both surgeon and patient [11,12].

In a study using intraoperative CT-based navigation for percutaneous iliosacral screwing, Peng et al. found markedly improved accuracy and reduced dependence on imaging compared with conventional fluoroscopy [10]. Our results corroborate these findings and demonstrate that such efficiency translates to SIJ arthrodesis as well. Although setup and registration steps might be expected to prolong procedures, we did not observe a significant increase in operative duration. The mean operative time tended to be shorter in the navigation group (146 ± 37 min vs 162 ± 31 min), suggesting that streamlined trajectory planning may offset system preparation. Comparable results were described by Shuman et al., who found that navigation did not significantly lengthen spinal procedures once the learning curve stabilized [11]. Likewise, Heydar et al. confirmed that operative time differences between navigation and conventional approaches become negligible after adequate experience [13].

A systematic review of navigation-guided spine surgery reported mean reductions in radiation dose of 20-60% compared with freehand fluoroscopy [14]. Radiation dose was lower in the navigation group (718 ± 379 mGy·cm²) compared with the non-navigation group (1015 ± 532 mGy·cm²), although the difference was not statistically significant. This finding is consistent with previous research showing that image-guided or robotic systems can reduce radiation exposure, although outcomes vary depending on case complexity and surgeon experience [15]. The modest reduction observed in our study likely reflects the limited sample size and the already low baseline exposure associated with a single-level procedure.

Intraoperative blood loss did not differ significantly between groups. Because both techniques require similar exposure and decortication, navigation is unlikely to influence bleeding directly. This observation aligns with prior spine literature showing comparable blood loss between navigation-assisted and conventional approaches [14,16]. Recent publications have extended navigation and robotic technology specifically to SIJ fusion. Chaves et al. reported excellent accuracy and low radiation with robotic navigation in 36 patients undergoing SIJ fusion [16]. Peterson et al. compared robotic and navigated SIJ fusions and found no difference in complication rate or accuracy, supporting the safety of both methods [17]. Collectively, these studies, along with our results, indicate that navigation improves imaging workflow and maintains comparable operative metrics and safety [18].

This study has several limitations. The small sample size (n = 30) limits statistical power and increases the risk of type II error. Its retrospective design introduces potential selection bias. Moreover, all operations were performed by a single senior surgeon, which ensures consistency but may introduce operator bias or learning-curve effects. Finally, clinical outcomes and fusion rates were not assessed, focusing solely on perioperative parameters. The significant reduction in fluoroscopy time suggests that navigation offers tangible benefits for radiation safety and workflow optimization, particularly in high-volume centers or cases requiring multiple imaging sequences. However, since operative time and dose were not significantly altered, the decision to adopt navigation should balance efficiency gains against cost, training, and equipment availability. Future multicenter prospective trials with larger cohorts should examine long-term outcomes, fusion rates, and cost-effectiveness to refine patient selection and maximize benefit from navigation systems.

## Conclusions

In this comparative analysis, navigation-assisted sacroiliac joint arthrodesis showed a meaningful reduction in fluoroscopy time without significantly affecting operative duration, radiation dose, or blood loss. These findings suggest that navigation may improve imaging efficiency and intraoperative workflow. While the results are encouraging, they should be interpreted with caution due to the small sample size and retrospective design. Larger multicenter studies with long-term follow-up are warranted to determine whether navigation provides measurable benefits in surgical precision, radiation safety, and patient outcomes.
